# Designing the Insulation System for Motors in Electrified Aircraft: Optimization, Partial Discharge Issues and Use of Advanced Materials

**DOI:** 10.3390/ma14247555

**Published:** 2021-12-09

**Authors:** Robin Ramin, Gian Carlo Montanari, Qichen Yang

**Affiliations:** Center for Advanced Power Systems, Florida State University, 2000 Levy Avenue, Tallahassee, FL 32310, USA; gmontanari@fsu.edu (G.C.M.); qyang@caps.fsu.edu (Q.Y.)

**Keywords:** partial discharges, insulation design, aircraft, motor, inverter-feeded machine, corona-resistant material

## Abstract

Designing the insulation system for motors to be used in electrical aircraft requires efforts for maximizing specific power, but, in parallel, particular attention to achieve high reliability. As a major harm for organic insulation systems is partial discharges, design must be able to infer their likelihood during any operation stage and handle their potential inception. This paper proposes a new approach to carry out optimized or conservative insulation system designs which can provide the specified life at the chosen failure probability as well as look at the option of possibly reducing the risk of partial discharges to zero, at any altitude. Examples of designing turn, phase to ground and phase-to-phase insulation systems are reported, with cases where the design can be optimized and other cases where the optimized design does not pass IEC testing standard. Therefore, the limits for design feasibility as a function of the required level of safety and reliability are discussed, showing that the presence of partial discharges cannot be always avoided even through conservative design criteria. Therefore, the use of advanced, corona-resistant materials must be considered, in order to reach a higher, sometimes redundant, level of reliability.

## 1. Introduction

**Figure 1:**
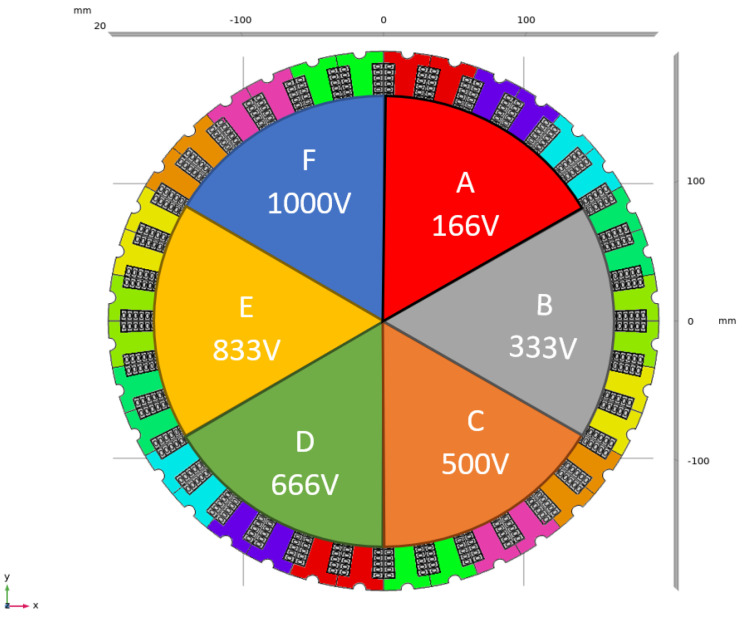
Scheme of the motor with its sections, each fed by a PWM supply, and the relevant nominal (maximum) voltage levels.

**Figure 2:**
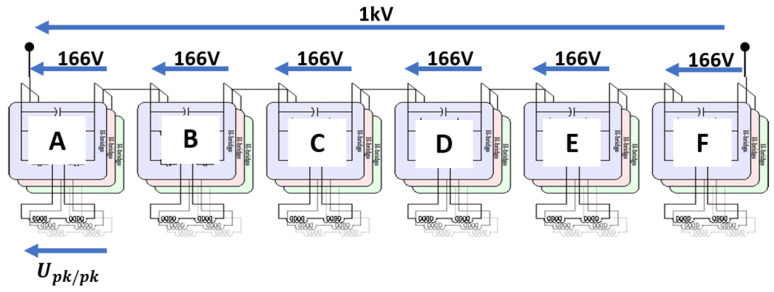
Scheme of the voltage potential distribution along the windings.

In order to show how the innovative design approach proposed here works and how this interlaces with the selection of insulating materials, a case based on a recent DOE ARPA-E project, ASCEND, is dealt with here. The project has the aim to design and manufacture a PWM-controlled motor for electrical aircraft, at a voltage up to 1 kV and power larger than 250 kW. The driving ideas is to have a motor divided in sections, each driven by its PWM control with 166 V. The conductors are cooled internally and made through additive manufacturing [1,2]. Figure 1 and Figure 2 sketch the machine structure with the sectors and relevant voltages. The insulation system design has to handle the turn-to-turn, the phase-to-ground and the phase-to-phase subsystems, taking into account the specified life, at the chosen failure probability, and the risk and impact on accelerated aging of partial discharge, PD, both at ground and at cruise air pressures. Insulating material would be organic, to try to maximize the specific power reducing insulation thickness and weight. Therefore, design, quality and acceptance testing should be covered by IEC 60034-18-41 [3], even if the nominal voltage would be higher than the limit considered by such standard. Accordingly, the insulation design should prove to be PD free up to test voltage levels that depend on the expected converter switching overshoots (encompassing stress categories from benign to extreme), aging and temperature effects [3,4,5,6,7,8,9,10,11]. In the specific case of this project, the very short distance between inverter and machine runs into the benign category, even if switching rise time is some tens of nanoseconds. Criteria for insulation design that can be able to provide the specified life and infer the likelihood of PD are provided in the next sections, highlighting conditions where the design can be optimized, i.e., involving a minimum volume of insulation, or conservative, that is, having the purpose, when feasible, of minimizing the likelihood of PD. The new idea named “three-leg approach” is developed in Section 2, applying it to the three insulation sub-systems in Section 3. Design quantities bringing to the choice if insulation thickness are discussed in terms of reliability and likelihood of PD inception, at different pressure as the aircraft can experience during take-off/landing and cruise.

## 2. Design Approach

**Figure 3:**
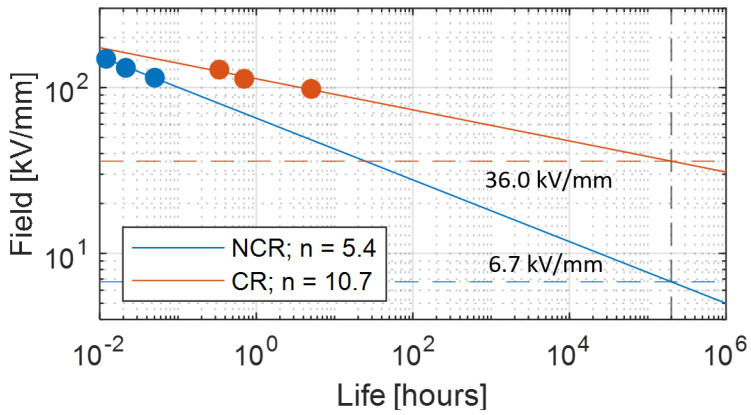
Life lines under PD and at failure probability 1% (Equation (1)) for non-corona-resistant material (NCR) and a corona resistant one (CR). The results of accelerated life tests (until breakdown) are reported, representing the failure time at failure probability 1% derived from the Weibull distribution of breakdown times (Equation (2)). The horizontal lines indicate the design field, $E_{D}$, corresponding to the life of $2\times 10^{5}\;$h (vertical dotted line) at failure probability 1%.

**Figure 4:**
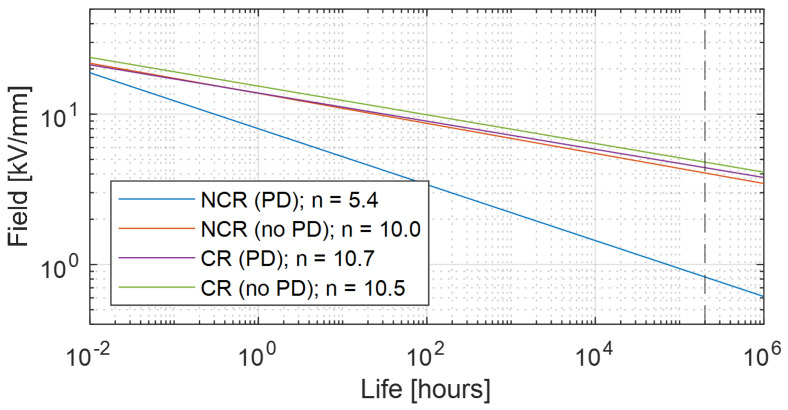
Life lines for CR and NCR materials, under PD (as in Figure 3) and without PD. The inverse of the slope (*n*, Equation (1)) drops down for NCR (from 10 to 5.4) when PD are active, but it remains almost constant (about 10) for CR materials. The design life, $L_{D}$, ($2\;\times \;10^{5}$ h) is indicated (vertical dotted line).
(iv) After the design is completed in terms of life and reliability specifications, the insulation system has to pass tests according to IEC 60034-18-41 [3]. This standard establishes test voltage values and procedures for design and qualification tests of rotating machines controlled by power electronics, introducing the IVIC (Impulse Voltage Insulation Class) concept. As regards test voltage values, Table 1 reports the limits obtained for benign and extreme stress conditions, mostly related to the extent of switching overshoots for the inverter components. As this depends on slew rate and length of cable connecting inverter to motor [3], the whole adjustable speed drive design considered in ASCEND (where inverters are connected directly to machine sections, Figure 1) brings test voltages towards benign stress conditions. The column of Table 1 related to insulation thickness to get a PD-free design will be commented later, when dealing with specifically the three insulation subsystems, that is, turn-to-turn, phase-to-ground and phase-to-phase. Calculations are based on a cavity/delamination thickness of 100 μm. The outcome of the test is that PD must not incept in the machine/insulation systems, thus the potential presence of defects able to generate PD must be accounted and weighted, in terms of reliability impact, at the design stage, in order to get the IEC test passed. Note that the insulation thickness design has nothing to do with the test voltage of Table 1, because it refers to nominal operating voltage. Indeed, the duration of such tests is so limited that even if the voltage exceeded the nominal one, their effect on aging rate is negligible.
(v) The actions that can be taken if the design is optimized and there is likelihood of PD inception in small cavities:
  - Use PD (corona) resistant material.
  - Considering thicker insulation distance to reduce the field overall and bring the field in defects below the PD inception value, Equation (5).
  - Implement Vacuum Pressure Impregnated (VPI) insulation to try to avoid cavities or delamination within insulation layers.
  The use of advanced corona resistant material is, however, always recommended, being a further level of redundancy as regards insulation system reliability.

**Figure 5:**
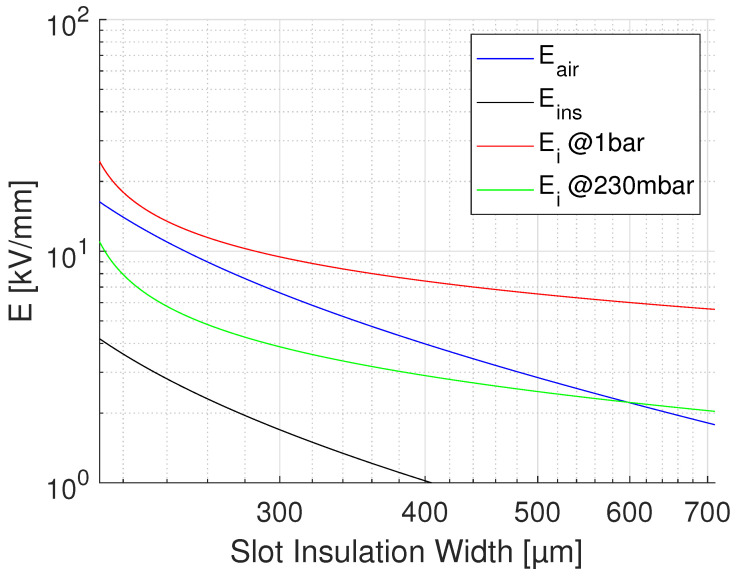
Behaviour of PD inception field, $E_{i}$, as a function of air gap thickness (thus slot width) in a slot filled by an insulation layer of 200 μm, nominal voltage 1 kV, $\epsilon_{rins}$ = 3.9. Ground air pressure, 1 bar, and 230 mbar. The field in air and in insulation, and the design field are also reported.

**Figure 6:**
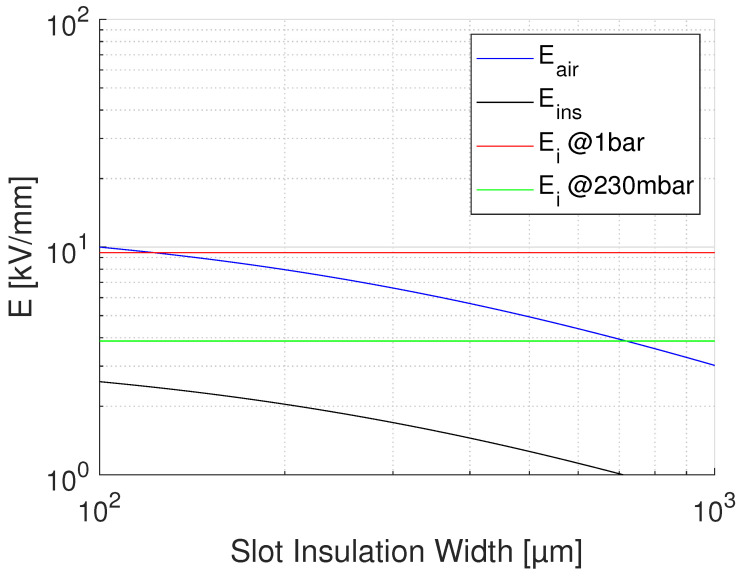
Behavior of PD inception field, $E_{i}$, as a function insulation thickness (thus slot width) with fixed air gap of 100 μm. Same parameters of Figure 5.

The design approach can be structured as follows:

(i) Design the insulation to last reliably, according to specifications, under nominal operation conditions.
  The design field can be determined extrapolating the results of accelerated electrothermal life testing carried out on candidate materials, and extrapolating the life lines to the design field and failure probability. For this purpose, the following expression, based on the generalized Weibull distribution, can be used to fit to failure times of accelerated life tests and suitable to be extrapolated to design life and stress [12,13]:
  (1) $$\log\left(t_{F,P}\right)=\log\left(K_{P}\right)+\log\left(\frac{f}{f_{0}}\right)+\log\left(t_{R}\right)-n\log\left(E\right)+\log\left(E_{R}\right)$$
  where $t_{F,P}$ is failure time, *E* is electrical stress, $K_{P}=-\left(\log{\left(1-P\right)}^{1/\beta_{t}}\right)$, *P* is failure probability, $\beta_{t}$ is the shape parameter of the Weibull function of failure times, *n* is voltage endurance coefficient, $E_{R}$ is reference electric stress (generally close to the electric strength), $t_{R}$ is failure time at applied field $E=E_{R}$, $f_{0}$ is the reference frequency (e.g., 50 or 60 Hz) and *f* the modulation/carrier frequency (depending on inverter level number) for AC modulated voltage. Note that the quantities in model Equation (1) depend on temperature, making Equation (1), as a matter of fact, an electrothermal life model. Figure 3 (partly taken from [14]) shows an example of life lines under PD for non-corona resistant (NCR) and corona resistant (CR) materials, that can be taped on the turn conductors or provide the slot insulation in the form of laminates.
  The results of accelerated life tests (till breakdown), under PD activity, are reported, representing the failure time at failure probability 1% derived from the Weibull distribution of breakdown times [15]:
  (2) $$P\left(t_{F}\right)=1-e^{-{\left(\frac{t_{F}}{\alpha }\right)}^{\beta }}$$
  where $t_{F}$ is failure (or breakdown) time, or life, and α and β are scale and shape parameter, respectively (the former can be expressed thus by Equation (1) [16]). The failure probability *P* = 0.01 corresponds to the life value extracted from each accelerated life test (dots in Figure 3). In the figure the design field, $E_{D}$, corresponding to the life of $2\times 10^{5}$ h (∼22 years) at failure probability 1% is reported. As can be seen, if PD are likely the NCR material can be used at design fields lower than 7 kV/mm, while the CR material can withstand a field $E_{D}\leq$ 36 kV/mm. Note that the life data in Figure 3 are relevant to accelerated life tests at 60 Hz (thus, the ratio of frequencies in Equation (1) is equal to 1). These results are for very thin specimens of limited dimensions. To go towards the design of a real insulation system, the dependence of breakdown strength and design stress on insulation thickness (and also length or surface) has to be taken into account. The larger the thickness and the longer or wider the insulation, the higher the probability to have weak points which can cause breakdown under electrothermal stress: this is called dimensional or size effect. It holds for electrical and mechanical stress because failure times fit to an extreme value distribution, as the Weibull function (Equation (2)). Attributing the subscript *R* to the quantities derived from tests, design failure time, $t_{FD}$, or life, $L_{D}$, for the real-size insulation system can be expressed (from Equations (1) and (2)) by
  (3) $$t_{FD}=t_{R}{\left(\frac{E_{R}}{E_{D}}\right)}^{-n}\frac{f_{0}}{f}{\left[\frac{\log(1-P_{D})}{\log(1-P_{t})}\right]}^{1/\beta_{t}}$$
  where $P_{D}$ and $P_{t}$ are design and reference (test) probability, respectively, and $E_{R}$, $E_{D}$ the test and the design field for the longer, larger or thicker insulation, respectively. Fixing design life, $t_{FD}$, the relationship between design and test (reference) field becomes
  (4) $$E_{D}=E_{R}{\left(\frac{l_{R}d_{R}}{l_{D}d_{D}}\right)}^{1/\beta_{e}}$$
  where $l_{R}$, $l_{D}$ are lengths of reference (test) and real-size insulation, respectively; $d_{R}$ and $d_{D}$ test and design thickness; and $\beta_{e}$ is Weibull shape parameter of electric strength distribution values. Applying Equation (4) to the results of accelerated life testing on CR and NCR materials, with a thickness going from 0.03 mm to 0.2 mm, and $l_{D}$ = 10$l_{R}$, and $\beta_{e}$ = 2 (conservative), the life lines of Figure 4 are obtained from those of Figure 3. As can be seen, for the CR material aged under PD the design field goes from 36 to about 4.5 kV/mm, for the design life of $2\times 10^{5}$ h. The design field of the NCR material would be 0.8 kV/mm, which makes it an inappropriate choice in the presence of extrinsic accelerated ageing caused by PD. In the absence of PD (see the lines of Figure 4, obtained performing accelerated ageing in oil), the design field of NCR goes up to about 4 kV/mm, while that of CR is near to 5 kV/mm. This clearly indicates that if the presence of PD is likely (and even if it is not, for the sake of redundancy in reliability) the choice of advanced CR materials is unavoidable. If the modulation frequency is different from that of the tests (60 Hz), life lines should be scaled down of a value equal to the frequency ratio, e.g., of 20times if the modulation frequency is 1200 Hz. This provides the same design, but for a life of $1\times 10^{4}$ h.
(ii) Calculate the insulation thickness, depending on design field and nominal voltage.
  The thickness depends on the criterion $E_{D}\leq$ maximum field in insulation. Actually, in the presence of significant electric field gradients it is not established yet whether reference should be made to the maximum or the mean field: the former will be certainly a conservative criterion, as local damage grows slower to breakdown than under the same field uniformly distributed (this is a topic that would require rapid and dedicated research work). Thickness can be managed to be as thin as possible, depending on the chosen material, thus on the maximum design field (e.g., for the CR material of Figure 4, this could be 4.5 to 5 kV/mm). However, thickness can be increased whether the driving criterion is to reduce the likelihood of PD inception in possible cavities or triple points: see next point.
(iii) Estimate the PD inception likelihood.
  Even if manufacturing should, in principle, avoid the presence of defects able to incept PD (as cavities, interfaces where low-density means are involved), taped insulation or cast resin can involve gas bubbles or interfaces between layers, and thermo-mechanical aging can create such defects where PD can be generated after some time of operation. A deterministic estimation of the field at which PD can be triggered can be roughly derived from the work in [17], that is
  (5) $$E_{i}=25.2p\left(1+\frac{8.6}{\sqrt{pd}}\right)$$
  where *p* is the pressure (in Pa) and *d* is the diameter (in m) of the cavity or cavity thickness. In the case of electrical aircraft motors, pressure must be varied down to that e.g., 23,000 Pa (230 mbar), corresponding to altitude of 35,000 ft.
  Note that the electric field does not change significantly (in terms of maximum value and profile) with AC voltage waveform (modulated or sinusoidal), with the exception of the extent of the transient following switching in power electronics supply. This, however, would not influence significantly life especially in the benign stress conditions dealt with here [18]. On the contrary, the partial discharge inception voltage, PDIV, (and field) is function of the rise time and number of inverter levels. However, this cannot be taken into account in the above model, considering that the inception field, $E_{i}$, is provided by a deterministic model and, on the other hand, this is already account for in the IEC PD tests (which can be done under sinusoidal voltage with peak value suitable increased). The difference in PDIV between sinusoidal and fast rise time voltage waveforms is provided by the stochastic component of PD events, mostly by the delay time of the first available electron and by the drifting/recombination of space charge deposited by each discharge. These phenomena can generally raise the PDIV [5,11,16,19,20]. Thus, the $E_{i}$ calculated in the paper can be considered a conservative estimate.
  Figure 5 shows, as an example, the behaviour of $E_{i}$ as a function of air gap thickness in a slot filled by an insulation layer of 200 μm, nominal voltage 1 kV, insulating material relative permittivity $\epsilon_{rins}$ = 3.9. The air gap is varied from 10 to 500 μm (thus slot width goes from 210 to 710 μm). The case of slot of variable width, fixed air gap of 100 μm and variable insulation width until the maximum field is $\leq E_{i}$ is reported in Figure 6. Both figures consider ground air pressure and at 230 mbar. Note that Figure 5 can represent the case of optimized design, where design field is maximum and insulation thickness minimum, in which the likelihood of PD at 1 bar is negligible, but PD can incept already for defects >10 μm at 230 mbar (up to an air gap of 600 μm). On the contrary, Figure 6 introduces to a conservative design where PD would not incept, at variable pressure and for an air gap of 100 μm. At a total slot insulation width of 720 μm (100 μm air and 620 μm insulation material) the electric field in air is lower than the inception field at 230 mbar. The choice of the design solution becomes aware, then, of the acceptable risk in relation to the specified life and failure probability. It is evident, looking at Figure 4, that if PD incept and act persistently, life sharply decreases, especially in an NCR material. Indeed, Figure 4 highlights that the voltage endurance coefficient, *n* (Equation (1)), drops down for NCR (from 10.0 to 5.4) when PD are active, but it is almost constant for CR materials (about 10).

**Table 1:** Test voltages for the three insulation sub-systems (see Section 3) and minimum insulation thickness where PD likelihood is estimated as negligible (Equation (3)) for benign and extreme test condition (based on IVIC concept in [3]). Sinusoidal or impulse voltage, same modulation frequency. Values between brackets correspond to a worst case of phase-to-phase insulation.

Passing without PD	Turn/Turn		Phase/Ground		Phase/Phase	
	Voltage	Ins. Thick. (H1)	Voltage	Ins. Thick. (H2)	Voltage	Ins. Thick. (H3)
	V	μm	V	μm	V	μm
Nominal Operating Conditions (Design Voltage)	150	43	1000	770	333 (1333)	110 (1200)
Benign Test Conditions	227	55	1287	1150	953 (1619)	700 (1650)
Extreme Test Conditions	529	250	1892	2070	(2621)	(3500)

## 3. Designing the Insulation System

**Figure 7:**
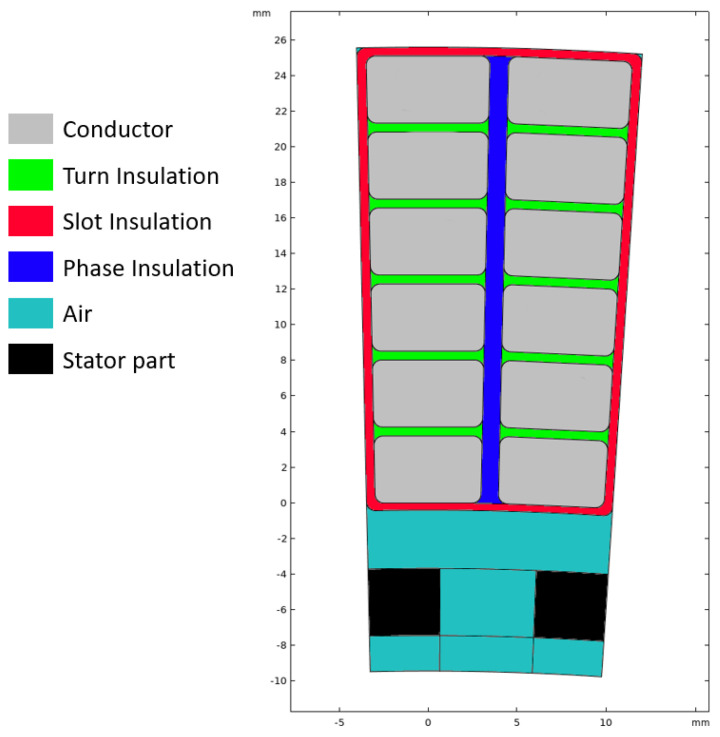
Drawing of the slot cut-through with windings of different phase. Turn-to-turn (green), phase-to-ground (red) and phase-to-phase (blue) insulation are indicated around the turns of the windings (grey).

Figure 7 sketches the structure of a slot with conductors and insulators, highlighting turn-to-turn, phase-to-ground (slot) and phase-to-phase insulation systems. Field simulations were done using COMSOL and a sinusoidal supply voltage at frequency 20 kHz. Using FEM (finite element method) simulation gives additional information on the field distribution, especially for non-uniform fields which are not accounted for in the analytic models described previously. Furthermore, additional environmental aspects can be considered (temperature, humidity, etc.). In the following, H1 is the turn-to-turn insulation thickness, H2 is the phase-to-ground insulation thickness and H3 is the phase-to-phase insulation. The reference design field, based on Figure 4, is taken 5 kV/mm (slightly higher than the 4.5 kV/mm provided by Figure 4 for CR material, but the plots of Figure 4 are very conservative having chosen a very small value of $\beta_{e}$ = 2).

### 3.1. Turn-To-Turn Insulation Design

**Figure 8:**
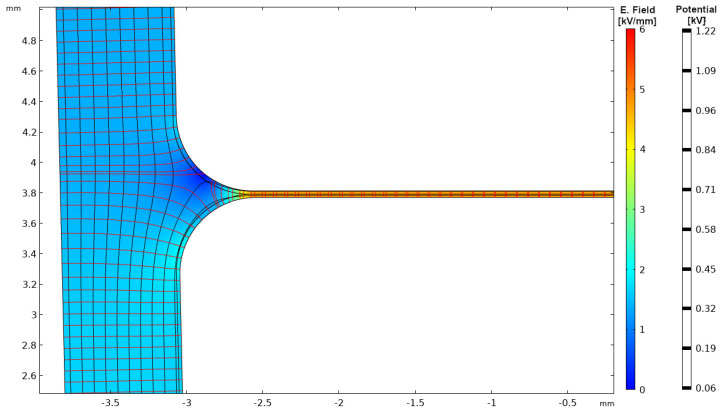
Electric field distribution in the slot considering the worst condition for the turn-to-turn insulation (benign condition). Only the turn-to-turn insulation applied in this figure, but no slot insulation.

Keeping in mind that the voltage drop, considering the very short switch time, is mostly on the first couple of turns [3], insulation design is related to the worst condition, that is to the first turns. There, the maximum voltage drop is 150 V (166.6 V × 0.9, Figure 2), while on the next turns it becomes negligible (see the example of Figure 8). As mentioned in the previous section the design field is chosen slightly higher than for the advanced CR material life line of Figure 4, the minimum insulation thickness (Figure 7) is H1 = 30 μm (150 V divided by 5 kV/mm), which corresponds to the optimum design (lowest insulation volume and weight).

**Figure 9:**
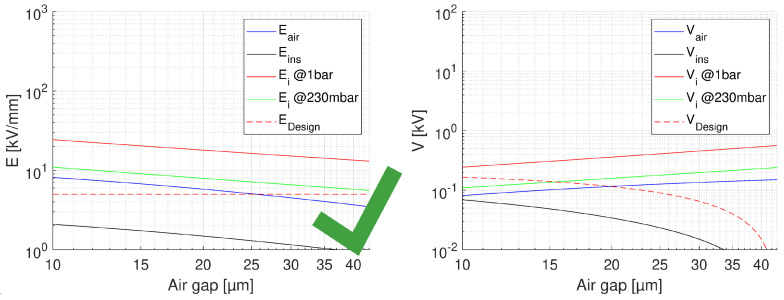
Feasibility plot for the optimum design of turn insulation, with the highest design field (5 kV/mm), and thinnest insulation (43 μm). Maximum voltage drop between turns 150 V. The x axis reports the air gap thickness. Left: field, Right: voltage. No PD will be incepted in cavities of any size and in the considered pressure range.

**Figure 10:**
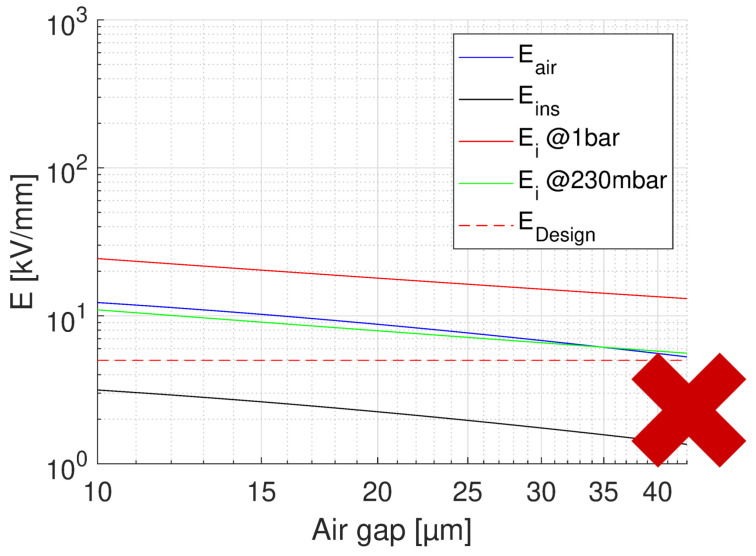
Feasibility plot for the optimum design of turn insulation, with the highest design field (5 kV/mm), and thinnest insulation (H1 = 43 μm). Maximum voltage drop between turns 227 V (benign test conditions, Table 1). The x axis reports the air gap thickness. PD will be incepted in cavities of any size above 10μm at 230 mbar (35,000 ft).

**Figure 11:**
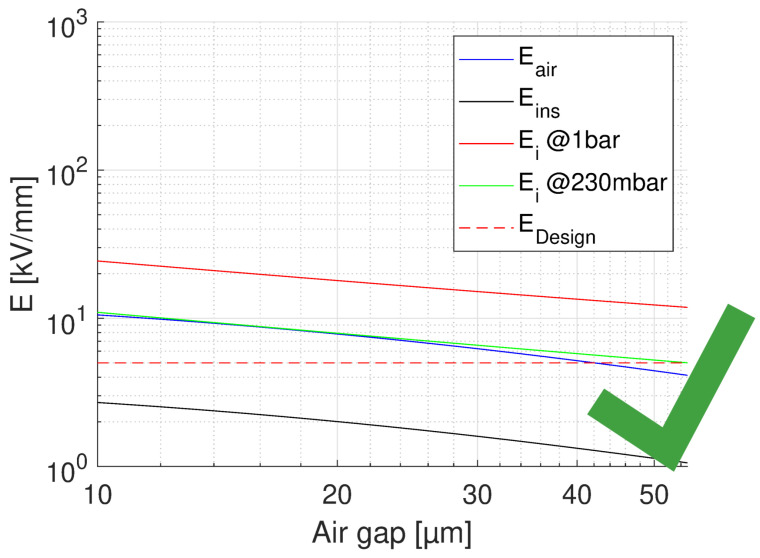
Feasibility plot for the design of turn insulation, with the insulation thickness (H1 = 55 μm) providing no PD for any air gap and considered pressure range. Maximum voltage drop between turns 227 V (benign test conditions, Table 1). The x axis reports the air gap thickness.

As regards PD inception estimate, the field in a cavity/delamination, $E_{air}$, having a given thickness, e.g., 50 or 100 μm, and in the insulation, $E_{ins}$, can be calculated by simulation or, approximately, resorting to the following relationship (valid for uniform-field geometry) [21,22]:

(6) $$E_{air}\;=\;\frac{V\epsilon_{ins}}{t_{air}\epsilon_{ins}\;+\;t_{ins}\epsilon_{air}}$$

(7) $$E_{ins}\;=\;\frac{V\epsilon_{air}}{t_{air}\epsilon_{ins}\;+\;t_{ins}\epsilon_{air}}$$

where *V* is nominal voltage and $t_{air}$, $t_{ins}$ are thickness of the air and insulation layer, respectively. The relative permittivity of the insulating material considered here (CR) is $\epsilon_{ins}$ = 3.9. A summary for the feasibility of turn-to-turn insulation, for the worst case of voltage drop (first turns) is provided by Figure 9, Figure 10 and Figure 11. In each figure, the field in air and insulation (Equations (4) and (5)), the design field (5 kV/mm), the PD inception field at ground and at 35,000 ft (Equation (3)) are reported. The feasibility condition is that the field in air is lower than the inception field at any considered pressure and that the field in insulation is lower than the design field. Fulfilling the latter condition would bring the specified life and reliability, while the former condition has to do with the likelihood of PD inception: $E_{air}\;<\;E_{i}$ would mean low PD likelihood. Figure 9 shows the feasibility for the optimum design, that having the highest design field (5 kV/mm), thus the thinnest insulation (H1 = 43 μm) and a maximum voltage drop between turns as that expected during operation, i.e., 150 V.

The x axis reports the air gap thickness, so that the total distance between two conductor turns is $t_{air}$+H1. As can be seen (Figure 9), no PD will be incepted in cavities of any size. Figure 9 supports the same approach referring to voltages, rather than fields. The same does not occur if we consider the testing condition, even for the benign stress level, see Table 1. The maximum voltage drop will be now 227 V, i.e., 252 × 0.9 V, which changes drastically the feasibility outcome, as seen in Figure 10. Above 10 μm PD will always be present. If we want to think of the limit condition which reduce the field in air gaps below the PD inception, as in Figure 9, insulation thickness must be increased, specifically above H1 = 55 μm. As displayed by Figure 11, PD will not be incepted for any cavity size and the turn-to-turn insulation system will likely pass the IEC tests [3]. Summarizing, the optimized design would point on H1 = 43 μm insulation thickness, but in the presence of cavities this solution may not pass the IEC test. Increasing insulation thickness to 55 μm would reduce insulation field and air gap field, so that the latter would be below inception field also under benign test conditions (as highlighted in Figure 6). The design will not be corresponding anymore to minimum insulation volume or weight, but it would strengthen machine reliability. In addition, since presence and size of defects cannot be forecasted, especially if caused by aging, the use of advanced dielectrics, as advanced CR insulating tapes, should become a fundamental requisite to support, even with redundancy, the reliability of a motor used in a such a critical asset as that of an electrical aircraft.

### 3.2. Phase-to-Ground (Slot) Insulation Design

**Figure 12:**
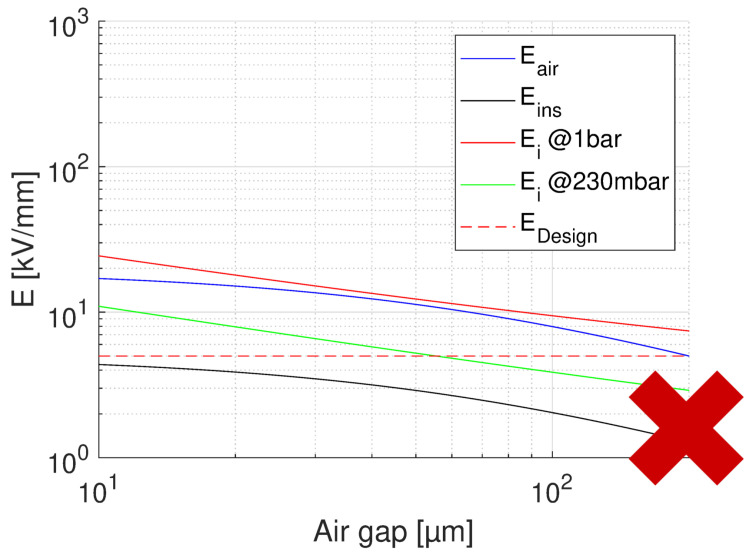
Feasibility plot for the optimum design of slot insulation, with the highest design field (5 kV/mm), and thinnest insulation (H2 = 200 μm). Nominal operating voltage 1000 V (Figure 2, Table 1). The x axis reports the air gap thickness. No PD will be incepted in cavities of any size at ground pressure, but PD will likely incept for cavities ≥10 μm at 250 mbar (35,000 ft).

**Figure 13:**
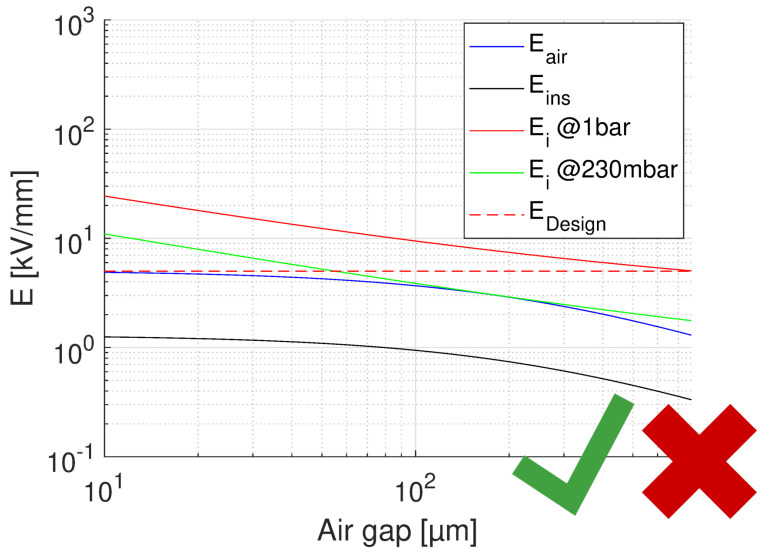
Feasibility plot for the design of slot insulation, with the insulation thickness (H2 = 770 μm) providing no PD for any air gap and considered pressure range. Voltage = 1000 V (Figure 2, Table 1). The x axis reports the air gap thickness.

**Figure 14:**
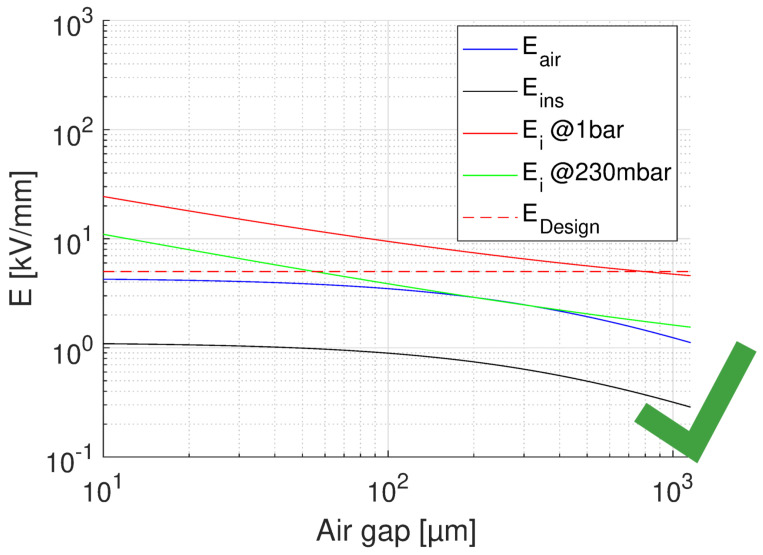
Feasibility plot for the design of slot insulation, with the insulation thickness (H2 = 1150 μm) providing no PD for any air gap and considered pressure range. Test voltage = 1287 V (Figure 2, Table 1). The x axis reports the air gap thickness.

The same type of insulating material, CR and having mechanically enhanced characteristics, or a laminate included in it, can be used as phase to ground insulation. The maximum phase to ground voltage under normal operation is 1000 V, Table 1. Based on field simulation and design field of 5 kV/mm, insulation thickness able to provide the specified life and reliability is H2 = 205 μm, which corresponds to the optimum design (lowest volume and weight). Feasibility plots for normal operation electrical stress and for testing (benign conditions) are shown in Figure 12, Figure 13 and Figure 14. Figure 12 indicates that feasibility with the optimum design, that is, maximum field and lowest thickness (H2 = 205 μm) does not provide PD at ground pressure, but PD may be expected during aircraft cruising.

Increasing insulation thickness to H2 = 770 μm, thus reducing insulation field, would provide a design PD free at any pressure in the considered range (see Figure 13), but this will not be valid anymore at the IEC test level, i.e., 1287 V (Table 1). In order to have a likely PD-free design able to pass the IEC test, an insulation thickness H2 = 1150 μm would be required for any cavity or delamination having thickness, see Figure 14. Decreasing cavity size, also the insulation thickness decreases, e.g., for expected air gaps not larger than 20 μm, the minimum PD-free insulation thickness becomes 430 μm for nominal conditions and 560 μm for the IEC test level. Summarizing, to turn insulation by contrast, the optimum design with H2 = 205 μm is capable to ensure the specified life, but most likely not the absence of PD at cruise air pressure. To go towards PD free design at nominal voltage (1000 V), an insulation thickness H2 = 770 μm would be needed for an air gap of 100 μm, while reducing the defect size (e.g., by more accurate and stable insulation taping or by resin impregnation) H2 diminishes, e.g., 600 μm for an air gap of 50 μm and 435 μm for an air gap of 20 μm. The thickness becomes even bigger in order to pass the IEC test, going up to 1150 μm for a cavity height of 100 μm, 820μm when considering cavities/delamination 50 μm thick, and 600 μm for an air gap of 20 μm. This would involve a sub-optimized design, with larger motor size and insulation volume and weight. As a matter of fact, either resin impregnation (VPI) or advanced CR materials, or, better, both would assist in obtaining the desired reliability with some level of redundancy. It can be speculated, indeed, that VPI can narrow down cavity size, below 20 μm. IEC PD tests can possibly confirm it.

### 3.3. Phase-to-Phase Insulation Design

**Figure 15:**
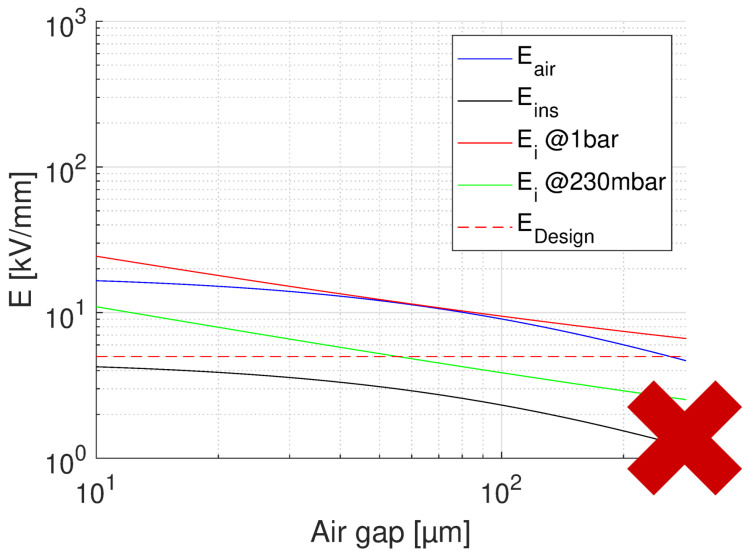
Feasibility plot for the optimum design of phase-to-phase insulation, with the highest design field (5 kV/mm) and thinnest insulation (H3 = 285 μm). Nominal operating voltage = 1333 V (Table 1). The x axis reports the air gap thickness. No PD will be incepted in cavities of any size at ground pressure, but PD will likely incept for cavities at 35,000 feet.

The same criteria as above will drive the design. Referring to Figure 7, H3 is the phase-to-phase insulation thickness. The summary of the results is reported in Table 1. They are relevant to both the optimized design based on the maximum field and to that relevant to test conditions. The optimized design provides an insulation thickness (cumulative of that for turns) H3 = 110 μm at nominal voltage 333V. However, the electrical field in air becomes higher than PD inception field when IEC test voltage is applied, i.e., 935 V, so that thickness must be raised to 700 μm to have a PD-free design at any altitude. There is also a worst case here, that is, when two phases are adjacent from stack A and F. In this case, the nominal operating voltage is 1333V and H3 = 285 μm for the optimized design. This design, however, does not indicate that operation at 35,000 ft is PD-free, as highlighted by Figure 15. To have a PD-free design at nominal operating voltage, insulation thickness must be raised to 1200 μm, as in Table 1, where the results of the worst case are reported in brackets. This becomes 600 μm when the cavity size is reduced to 20 μm. Such design will not pass IEC tests, for which an insulation thickness of 1650 μm would be required with a cavity height of 100 μm (745 μm if height is 20 μm). Summarizing, as for slot insulation the insulation thickness required to have a phase-to-phase PD-free design under IEC tests might be too large for a reliable, but size-constrained, design. The event to have coils from stacks A and F adjacent should be avoided; otherwise, resin-impregnation seem to be the realistic solution to bring to a reliable and PD-free design. In any case, advanced corona-resistant materials shall be used to keep an acceptable level of reliability and redundancy.

## 4. Conclusions

The basis criterion for the design of motors for electrified aircraft must be reliability. Since the major cause of accelerated degradation and premature failure of insulation systems (organic) under electrothermal stress is partial discharges, the design should be PD free. Furthermore, it must be PD free not only at nominal operating voltage, but also at the test voltage indicated by IEC 60034-18-41. To achieve this goal, the maximum size of cavities/delamination that may be present due to manufacturing or multi-stress aging should be controlled and limited, and insulation thickness increased from that corresponding to the optimum design under nominal operating conditions, so that the field in air gaps can go down the theoretical inception field. If this brings to unrealistic values of insulation thickness, solutions (as resin impregnation) must be adopted to reduce drastically cavity size. Note that only the benign conditions of electrical stress have been considered here to check for PD inception likelihood. Referring to extreme conditions would not allow any PD free insulation design with reasonable insulation thickness. The only feasibility option will become high-technology impregnation to remove any chance of cavities/delamination, also due to aging, with height larger than 10μm. Furthermore, frequent diagnostic PD testing or PD monitoring is needed to assess the health index as a function of operation time. In any case, the use of advanced corona (PD)-resistant materials for insulation design and manufacturing, which would provide a further level of redundancy in insulation system reliability, must be recommended if not mandatory.
